# On the Use of Antim. Tart. in Ophthalmia

**Published:** 1814-05

**Authors:** 


### *For the Medical and Physical Journal.*

### *On the Use of Antim. Tart. in Ophthalmia.*

NO sooner had I read the paper in the last Number of this Journal, signed "*An Admirer of Truth and Justice,*" than I immediately exclaimed—Wonderful man!

"——— Quæ te tam læta tulerunt

"Secula? qui tanti talem genuere parentes?"

But what has the author of this long paper in view? Does he either prove or disprove the opinions which I ventured to offer? I do not see that he even attempts it.

That the case which I related was the real Egyptian ophthalmia, I will not contend for; but I must remark, by the way, that there was considerable chymosis in that case, and when first seen a puriform discharge from the conjunctiva, which afterwards was succeeded by the effusion of tears; but, however, admitting that it really was not, he cannot deny that it was a most violent attack of inflammation, approaching to the degree of the Egyptian ophthalmia.

The treatment of Egyptian ophthalmia, and the simple acute ophthalmia, has always been similar, and the *modus operandi* considered the same; only that in the former the symptoms being more violent, the remedies employed have been carried to a greater extent. Now Mr. Adams, if I am not mistaken, (I have not the paper before me,) does not attempt to give a solution of any peculiar operation of the Ant. Tart. in that disease, but goes on general principles; 'tis true he did not say that he had tried it in the acute ophthalmia, but not giving the least intimation that he thought it would not be successful in that disease, and reasoning that if vomiting was so peculiarly successful in abating the inflammation of Egyptian ophthalmia, it would not fail, given in a similar manner, to be useful in acute ophthalmia, where the inflammation exists in a less degree; and that it was given in the manner directed by Mr. Adams, I must unquestionably maintain. It is quite immaterial whether he directs two grains or ten grains, a point which the gentleman insists on much; his only meaning in that is, that vomiting should be produced and continued; if, then, one grain would have that effect, where was the necessity of giving more until required? He seems wonderfully desirous to enforce the necessity of doing just as Mr. Adams says, and in a very eloquent passage shows the grand mistake I have fallen into in not attending more carefully to that circumstance, of which this sentence forms a part: "Mr. Adams, in his first paper on the Ophthalmia, inserted in the 170th

Number of this Journal, p. 303, after mentioning that the mode of treatment recommended by a celebrated oculist had been ineffectually tried by himself and his son, for the space of two years, upon a large number of patients, in St. Pancras workhouse, where he first tried his practice, says," &c. This is as clear and precise as the most critical could require; not any doubt or uncertainty arises on reading over the passage, who it was that tried the practice two years; one of those "who are so blind as will not see," perhaps might be at a loss; but those who have their eyes enlightened, will give him due praise for his *non-ambiguity*.

The assertion that I said the inflammation had pre-existed three days in the *most violent degree*, I must beg leave to say is not strictly just: I said that it had *commenced* three days previously, but that it had existed from the beginning in the most violent degree I did not say; for, in fact, the inflammation had only begun to be alarming the same day on which he was seen. Along with him two or three of his children were affected with considerable inflammation and puriform discharge from the eyes; which circumstance would favour the opinion that his case was Egyptian ophthalmia, it being contagious; but in them the inflammation did not extend to so violent a degree as to require such active remedies.

*Hull, March* 9, 1814. W. M. T.

